# A Full Green, Sustainable Paper-Based Packaging Material with High-Strength, Water Resistance, and Thermal Insulation

**DOI:** 10.3390/polym17010006

**Published:** 2024-12-24

**Authors:** Yongsheng Gu, Fengbiao Yao, Ruizhi Gong, Yong Di, Vennila Srinivasan, Xiaojie Hu, Baoxuan Liu, Dexiu Min, Chenglong Lian, Xiaoying Dong, Yongfeng Li

**Affiliations:** 1 Key Laboratory of State Forestry Administration for Silviculture of the Lower Yellow River, College of Forestry, Shandong Agricultural University, Tai’an 271018, China; guyongsheng1232021@163.com (Y.G.); fengbiaoyao@163.com (F.Y.); ruizhigong789@163.com (R.G.); vennilasrinivasan93@sdau.edu.cn (V.S.); huxiaojie918@126.com (X.H.); 2 Key Laboratory of Agricultural Film Application of Ministry of Agriculture and Rural Affairs, College of Chemistry and Material Science, Shandong Agricultural University, Tai’an 271018, China; 3 Taian Cellulose Ether Technology Co., Ltd., Tai’an 271000, China; diyong8@163.com; 4 Shandong Cork Oak Industrial Technology Research Institute Co., Ltd., Jining 272100, China; liubaoxuan123@126.com; 5 Shandong Xingang Enterprise Group Co., Ltd., Linyi 276013, China; mindexiu@126.com

**Keywords:** nanocellulose, cork, green packaging material, thermal insulation, water resistance

## Abstract

Paper-based packaging materials have gained attention from academia and industry for their outstanding environmental sustainability advantages. However, they still encounter major challenges, such as low mechanical strength and inadequate functionality, hindering the replacement of unsustainable packaging materials. Inspired by the remarkable strength of trees provided by cellulose fibers and the water and heat protection of trees provided by bark, this study developed a new biomass-based packaging material (SNC-C) that combines strength, thermal insulation, and water resistance. The material was created by simply blending straw nanocellulose (SNC) with oak bark (i.e., cork), which naturally provides water-resistant, thermal insulation, and unique regenerative properties. The dense layered structure formed entirely by SNC generates a tensile strength reaching up to 60.93 MPa. With the cork cavity structure, the heat transfer rate of the obtained material is reduced to 2.90–3.01 °C/(cm·min). The combining of the closed-cell structure and the suberin component of the cork results in a low water vapor transmission rate (WVTR) of the material of 400.30 g/(m^2^·24 h). This all-biomass material with excellent performance and low environmental footprint offers a promising solution for the development of sustainable multifunctional packaging materials.

## 1. Introduction

Packaging materials are essential for storing and protecting items from external environmental influences and play a crucial role in the transportation, exchange, and storage of goods [1,2]. These materials, primarily composed of plastic, cardboards, metal, and glass, have a global market size estimated at approximately USD 1.17 trillion, significantly contributing to economic and social development [3]. Notably, cardboard, which constitutes only 30% of the market, is the only packaging material with green recycling properties [4]. In light of the current global challenges such as resource depletion and environmental pollution, the green recycling of packaging materials is essential for promoting the sustainable development of the global economy and society [5,6]. Therefore, exploring and utilizing versatile paper-based packaging materials is desirable.

However, conventional paper-based packaging materials derived from cellulosic raw materials are typically weaker than those made from non-renewable resources in terms of mechanical and barrier properties [7,8]. For instance, in the cold-chain commodity economy, packaging materials must not only have high strength to support load-bearing requirements but also provide effective water vapor and heat barrier performance. Unfortunately, pure paper-based packaging materials, which exhibit poor water vapor barrier properties, often fail to meet these functional requirements due to diminished strength and reduced thermal insulation after exposure to water [9,10,11]. To improve these properties, modifications such as reinforcements, plastic diaphragms, and hollow structures are typically incorporated into paper-based packaging materials to enhance their mechanical strength, waterproof capabilities, and thermal insulation properties, respectively [12,13]. However, integrating all these properties can complicate the preparation process, and may compromise the sustainable properties of paper packaging materials [14]. For example, the introduction of nano-montmorillonite in cellulose matrix, combined with techniques such as foaming and hydrophobization, has enabled the production of paper-based packaging materials with good strength, effective water resistance, and heat insulation. Unfortunately, this approach also introduced non-renewable raw materials and complicate the preparation process [15]. The application of a chitosan/gelatin/microsphere aerogel coating on the surface of corrugated paper packaging materials can enhance the mechanical strength, water resistance, and thermal insulation properties of cardboard concurrently. However, it is important to note that this method relies on non-renewable raw materials for the microspheres [16]. Therefore, it is vital to focus on maximizing the use of renewable resources and developing green paper-based packaging materials that possess high strength, waterproofing, and thermal insulation capabilities through simpler process technologies. This endeavor is significant for meeting the diverse demands for packaging applications and promoting the sustainable use of such materials. However, to the best of our knowledge, there is no report on such materials. Therefore, the development of the materials presents both promising opportunities and considerable challenges.

**Figure 1:**
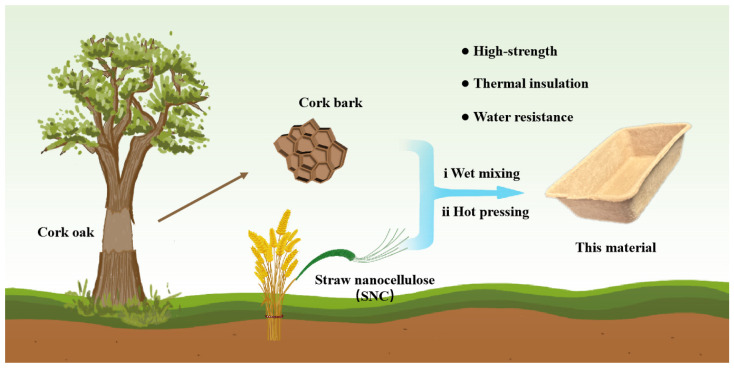
Schematic illustration of fabrication of the SNC-C.

The exceptional strength of trees is primarily attributed to the cellulose fibers [17], while the bark provides essential protection against water and aids in thermal insulation for the trunk [18]. Inspired by the structure of trees, this study introduces an innovative approach that employs a straw nanocellulose (SNC) to be physically blended with tree bark and hot-pressed to create an eco-friendly paper packaging material (see schematic diagram in Figure 1). It is worth emphasizing that the bark used in this study is derived from the cork oak tree, also known as cork, which possesses the unique sustainable property of being stripped and regenerating without affecting the growth of the trees [19,20,21]. Simultaneously, the unique honeycomb closed-cell structure of cork endows itself with a natural barrier against water and heat [22]. Therefore, this study introduces cork bark into the cellulose matrix without utilizing any complex pore-making processes such as foaming and freeze-drying, resulting in a green packaging material (SNC-C) using simple processes and simultaneously enhancing its thermal insulation and water-resistant functions. The final material demonstrated superior mechanical strength, excellent water vapor barrier property, and outstanding thermal insulation. We also investigated the interconnections among material components, structures, and properties. This study is anticipated to provide innovative design strategies for the development of multifunctional and environmentally friendly paper-based packaging materials.

## 2. Materials and Methods

### 2.1. Materials

Wheat straw, with a density of 0.26 g·cm^−3^ and a moisture content of 7%, was obtained from the experimental field of Shandong Agricultural University. Cork particles, with a particle size of 100 mesh, a density of 0.16 g·cm*^−^*^3^, and a moisture content of 4.86%, were provided from Shandong Laucork Development Co., Ltd. (Yanzhou, China). The chemicals utilized during the extraction such as sodium hypochlorite solution (NaClO) and anhydrous ethanol (EtOH), all analytical grade, were purchased from Tianjin Kaitong Chemical Reagent Co., Ltd. (Tianjin, China) and directly used without any purification. As a control, commercially available wood cellulose-fiber paper lunch boxes, with a thickness of about 0.3 mm and a density of 0.59 g·cm^−3^, were purchased from Jiangyin Zhutang Qingbird Packaging Material Factory (Jiangyin, China).

### 2.2. Method

#### 2.2.1. Preparation of Straw Nanocellulose

Straw nanocellulose (SNC) was prepared using the mechanical method previously established by our research group [23]. Briefly, 35 g of chopped wheat straw was treated twice with 500 mL of sodium hypochlorite solution for 6 h each time, under light-free conditions, to remove the lignin and other impurities. The resulting fiber product was thoroughly washed with anhydrous ethanol and deionized water, until the solution became neutral. Next, the purified straw fiber was dispersed in deionized water to form an isolate straw cellulose (SC) with a concentration of 1 wt%, using a high-speed disperser. Finally, the dispersed straw cellulose was subjected to nanofibrillation using a high-pressure homogenizer (APV-200, SPX FLOW, Unna, Germany) at a homogenization pressure of 400 bar, and the process was repeated for 8 homogenization cycles, resulting in the production of an SNC suspension at a concentration of 1%.

#### 2.2.2. Preparation of SNC Boards

A 1% suspension of SNC was dried in an oven at 60 °C to volatilize the water. During this period, without stirring, the weight was recorded every hour until the final concentration of SNC hydrogel was 5%. This hydrogel was subsequently placed into a mold and subjected to a hot press molding process under specific conditions: a pressure of 2 MPa, a temperature of 60 °C, and a duration of 6 h. Afterward, the mold was allowed to cool naturally, before being cold pressed overnight, resulting in SNC boards with dimensions of 90 mm x 90 mm x 0.13 mm. The fabricated SNC boards were further maintained in an environment at a temperature of 25 ± 2 °C and a relative humidity of 65 ± 5% RH for 24 h before performance testing. For comparative analysis, straw cellulose boards (SC boards) were produced using the same methodology employed for the SNC boards.

#### 2.2.3. Preparation of Straw Nanocellulose/Cork Composite Boards

The appropriate proportions of 1% SNC suspension and cork particles were thoroughly blended using a high-speed mixer operating at 1000 rpm for 10 min. Subsequently, following the same preparation procedures employed for the SNC boards, we obtained the straw nanocellulose/cork composite boards (SNC-Cs) with dimensions of 90 mm × 90 mm × 0.3 mm. According to the study protocol, the mass fractions of cork particles were set at 20 wt%, 30 wt%, and 40 wt%, resulting in three distinct SNC-C composites designated as “SNC-C_20_”, “SNC-C_30_”, and “SNC-C_40_”, respectively.

#### 2.2.4. Characterization of Atomic Force Microscopy (AFM)

The microstructure of the specimens was observed using an atomic force microscope (AFM) (FM-Nanoview1000, Suzhou Flyingman Precision Instruments, Suzhou, China). The frequency range was established between 184 and 187 Hz, with a scanning rate of 0.5 Hz.

#### 2.2.5. Characterization of Scanning Electron Microscope (SEM)

The microstructure of both the surface and interior of the specimen was observed using a scanning electron microscopy (SEM) (JSM-6610LV, JEOL, Tokyo, Japan) with an accelerating voltage of 10.0 kV.

#### 2.2.6. Characterization of Transmission Electron Microscopy (TEM)

Approximately 10 µL of the SNC suspension was deposited onto a carbon-coated copper grid and then subjected to staining with ruthenium vapor for 3 min. The microscopic morphology of the specimens was subsequently investigated using a transmission electron microscope (Talos L120C, Thermo Fisher, Waltham, MA, USA) at an operating voltage of 100 kV.

#### 2.2.7. Characterization of FTIR Scanning

The functional groups present in the specimens were analyzed using a Fourier transform infrared spectrometer (Nicolet 5700, Thermo Fisher, Waltham, MA, USA) to evaluate changes in elemental species and chemical composition. Spectra were recorded over a range of 4000 to 400 cm^−1^, with a resolution of 4 cm^−1^ and an average of 32 scans.

#### 2.2.8. Mechanical Properties

In accordance with GB/T 1040.1-2006 “Plastics-Determination of tensile properties”, the tensile strength and elongation at break of the specimens were evaluated utilizing an electronic universal testing machine (UTM2502, SUNS, Shenzhen, China) [24]. The dimensions of the specimens were standardized at a length of 50 mm and a width of 10 mm, with a tensile rate set at 10 mm/min. Each property was evaluated through three parallel tests, and the average value was subsequently computed.

#### 2.2.9. Density Characterization

We tested the density of the sample according to the method reported in the literature [25]. The thickness of the board was taken as the average of six points measured by a micrometer caliper. The density was calculated by measuring the average dimensions and weights of the specimens.

#### 2.2.10. Water Vapor Transmittance Rate Experiment

A water vapor transmission rate tester (W3-031, Labthink, Ji’nan, China) was employed to assess the water vapor transmission rate (WVTR) of the specimens. The testing procedure followed the weight loss method, which included a 1 h warm-up period, an experimental temperature of 38 °C, and a relative humidity of 90%. The measurements were performed in triplicate, and the average value was subsequently calculated.

#### 2.2.11. Water Contact Angle

We assessed the water contact angle of the specimens following the methodology described in the existing literature [26]. The water contact angle was measured at five distinct locations on the surface of the specimen using 5 µL of deionized water as the probe liquid, with an optical contact angle measurement device (OCA15EC, Data Physics, Baden-Württemberg, Germany). Following this initial measurement, the water contact angle was re-evaluated at the same locations at different times, with measurements taken every five minutes. The average of the recorded values was subsequently calculated to determine the surface water contact angle at each specified time point.

#### 2.2.12. Thermal Insulation Property

The temperature variations of the specimens were evaluated at room temperature (25 °C) using an infrared imaging tester (DRH-II, Shanghai Biaozhuo Scientific Instrument, Shanghai, China). The samples, cut into 50 mm squares, were placed on two distinct temperature sources: ice, serving as a cold source at −15 °C, and a thermostatic heating plate, functioning as a heat source at 60 °C. The experiment commenced with the specimen placed on the surface of the thermal source and maintaining it for one minute. During this interval, the initial surface temperature of the specimen was recorded, along with the temperature after one minute had elapsed. These data will facilitate the calculation of the heat transfer rate of temperature difference per unit of time and thickness, enabling an assessment of the thermal insulation performance of the specimen.

## 3. Results

### 3.1. Morphological and Mechanical Analysis

**Figure 2:**
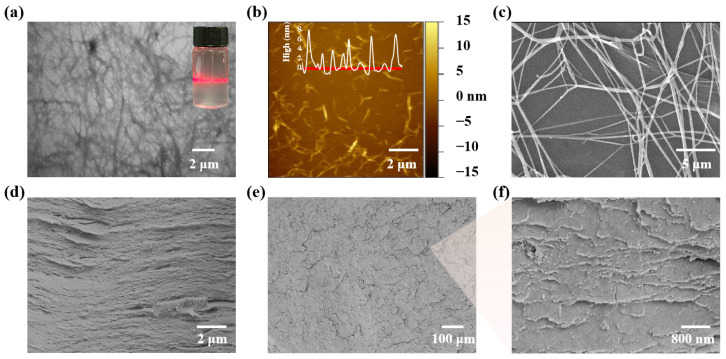
Structural images of straw nanocellulose boards. (**a**) TEM morphology (the upper-right inset shows a digital photograph of an aqueous suspension showing the Tyndall effect), (**b**) AFM morphology (the upper inset reflects the diameter of the SNC in the position of the red scanning line), and (**c**) SEM morphology of SNC. (**d**) Cross-sectional SEM morphology, (**e**) SEM morphology of the inner surface of SNC board, and (**f**) the magnified SEM morphology of (**e**).

Sequential pretreatment and homogenization of wheat straw resulted in an aqueous suspension of cellulose particles, which clearly exhibited the Tyndall effect when exposed to a laser pointer (inset in Figure 2a), indicating that the cellulose particles were in the nanoscale range. TEM morphology characterization confirmed that the resulting SNC comprised slender filaments with micrometer scale lengths and nanoscale diameters (Figure 2a), consistent with the Tyndall phenomenon. AFM morphology characterization further revealed that the length of the SNC fibers was ~1.5 μm, and the diameter was ~6 nm (Figure 2b, the upper inset of Figure 2b and Figure S1); thus, it can be evident that the resulting SNC has a high aspect ratio of ~250.

During the de-watering and drying process, the suspended SNC fibers in water self-assembled into high-aspect-ratio fibers with diameters of several hundred nanometers and lengths of several tens of micrometers, entangling with each other in a network-like fashion (Figure 2c), which contributes to the high mechanical strength of nanocellulose [27]. Notably, under the influence of external mechanical pressure during the de-watering and drying process, the SNC-derived network formed a dense lamellar structure, resembling the layered microstructures of shells (Figure 2d–f and Figure S2), suggesting that SNC boards have the potential to exhibit both high strength and toughness similar to natural shells [28,29].

**Figure 3:**
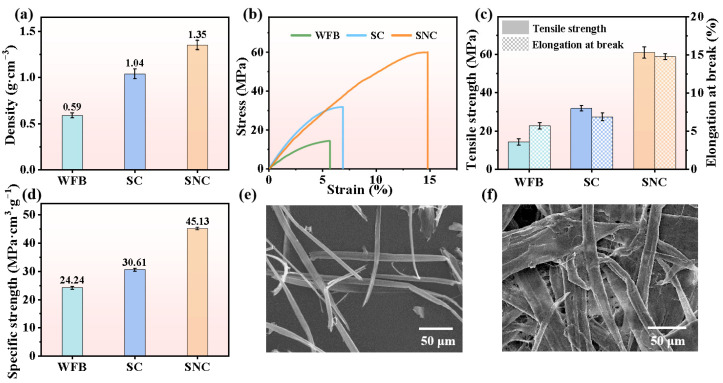
Mechanical properties of straw nanocellulose boards. (**a**) Density, (**b**) stress–strain curves, (**c**) tensile strength and elongation at break, (**d**) specific tensile strength of commercially available wood cellulose-fiber paper lunch box (WFB), SC board and SNC board, (**e**) SEM morphology of SC fibers, and (**f**) SEM morphology of fibers of WFB.

The dense internal structure of SNC boards resulted in a high density of 1.35 g·cm^−3^ (Figure 3a), which is 29.8% and 125% higher than that of mechanically untreated straw cellulose boards (SC,1.04 g·cm^−3^) and ordinary paperboards (commercially available wood cellulose-fiber paper lunch boxes, WFB, 0.60 g·cm^−3^), respectively. Remarkably, the tensile strength of the SNC boards reached 60.93 MPa, representing a substantial increase of 91.4% and 326% compared to the SC boards (31.83 MPa) and WFB (14.30 MPa), respectively (Figure 3b,c), demonstrating exceptional mechanical strength. Additionally, the elongation at break of SNC boards reached 14.78%, marking an improvement of 144% and 159% compared to SC boards (6.90%) and WFB (5.70%), respectively, showcasing remarkable toughness properties (Figure 3b,c). These outstanding toughness results align with the performance inferences derived from the aforementioned structural analysis.

The higher specific surface area and aspect ratio of SNCs lead to an increased inter-fiber entanglement force during the self-assembly process [25]. Simultaneously, the dense structure further enhances the inter-fiber hydrogen bonding and van der Waals forces [30]. The combined effects contribute to the high tensile strength of SNC boards (Figure 3b–d). Furthermore, the relatively weak interaction forces at the lamellar interface allow for dynamic slipping of the fibers under tension [31], as evidenced by the ductile tearing morphology of the fibers on the fracture surface (Figure S3), thereby imparting a high tensile elongation at break to the material. In contrast, pre-treatment on straw yields SCs with lengths of several hundred micrometers to 1 mm and diameters of approximately 10 μm (Figure 3e). As structural units constituting paper, these dimensions are only slightly smaller than those found in ordinary paper fibers (like the fibers of WFB) [32], but significantly larger than SNC sizes. The presence of such large-sized structural units results in more pores during assembly due to a smaller specific surface area (Figure 3f), leading to lower density (Figure 3a) and weaker interaction forces between structural units; consequently, SC and WFB materials exhibit weaker tensile strength. Comparatively, SC has slightly smaller structural units than ordinary paper fibers and marginally higher paper density (Figure 3a), and thus presents higher tensile strength than the ordinary paper (Figure 3b–d).

In conclusion, the SNC boards demonstrated superior mechanical properties (strength and toughness) that significantly surpassed those of conventional packaging paperboards, suggesting that abundant nanocellulose holds substantial potential for widespread application in sustainable packaging materials. This finding is pivotal for advancing the alternative utilization of agricultural and forestry waste resources in the realm of non-sustainable packaging materials.

### 3.2. Cork Introduced into SNC Boards to Enhance Thermal Insulation Property

**Figure 4:**
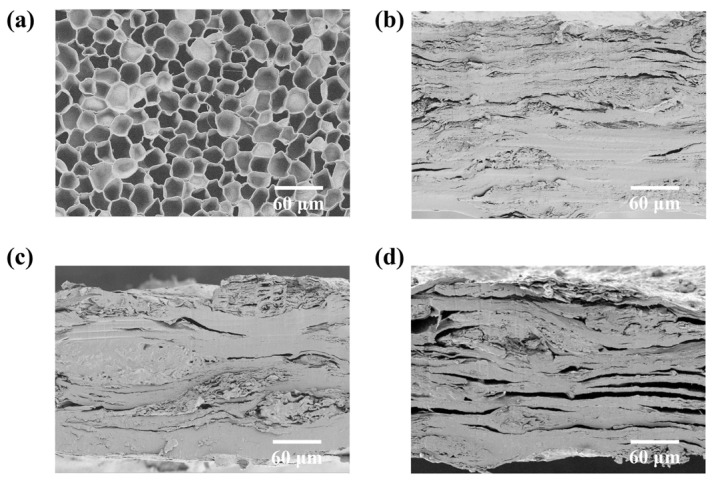
Structure of SNC-C. (**a**) SEM morphology of natural cork. (**b**) SEM morphology of cross-section of SNC-C_20_. (**c**) SEM morphology of cross-section of SNC-C_30_. (**d**) SEM morphology of cross-section of SNC-C_40_.

**Figure 5:**
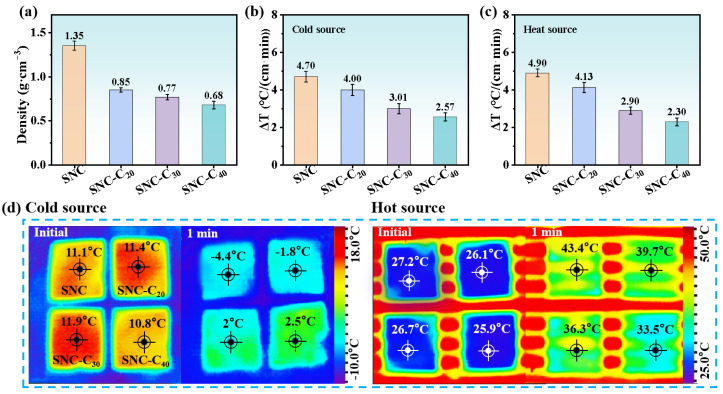
Thermal insulation of SNC-C. (**a**) Density, (**b**) heat transfer rate of temperature difference from cold, (**c**) heat transfer rate of temperature difference from hot source, and (**d**) infrared imaging photographs of heat transfer temperature from cold or heat sources of SNC-C with varying cork content.

Cork, a structurally unique bark, consists of hexagonal prismatic closed cells with an approximate diameter of 20 μm (Figure 4a) [33]. It exhibits an exceptionally low density of 0.16 g·cm^−3^ and possesses natural thermal insulation properties [34,35]. The incorporation of cork particles into the SNC boards results in the development of SNC-C composite packaging material and, accordingly, transitions the internal structure from a dense configuration (Figure 2d) to a porous arrangement (Figure 4b–d). At a cork content of 20%, the cross-sectional morphology clearly exhibits the cellular cavity microstructures introduced by cork particles, with slight cracks appearing at the cellulose lamellar interface (Figure 4b). These micro-pores reduce the material’s density from 1.35 g·cm^−3^ (Figure 3a) to 0.85 g·cm^−3^ (Figure 5a). As the cork content increases to 30% and 40%, the material’s internal porosity and interfacial cracks expand (Figure 4c,d), and the density correspondingly reduces to 0.77 g·cm^−3^ and 0.68 g·cm^−3^ (Figure 5a), respectively. These values approach the density of WFB (0.59 g·cm^−3^) (Figure 3a), aligning the material more closely with the requirements of the paper-based packaging material market.

Generally, a material with lower density and a higher number of internal pores exhibits more excellent thermal insulation properties [36]. Heat transfer experiments from cold sources indicate that the temperature change rates for SNC-C_20_, SNC-C_30_, and SNC-C_40_ were recorded at 4.00 °C/(cm·min), 3.01 °C/(cm·min), and 2.57 °C/(cm·min), respectively, representing reductions of 14.89%, 35.95%, and 45.32% compared to the pure SNC boards, which have a heat transfer rate of 4.70 °C/(cm·min) (Figure 5b–d). Similarly, in heat transfer experiments with hot sources, the temperature change rates for SNC-C_20_, SNC-C_30_, and SNC-C_40_ were recorded at 4.13 °C/(cm·min), 2.90 °C/(cm·min), and 2.30 °C/(cm·min), respectively, reflecting reductions of 15.71%, 40.82%, and 53.06% relative to the pure SNC boards, which exhibit a rate of 4.90 °C/(cm·min) (Figure 5b–d). These results indicate that SNC-C materials show notable heat barrier performance, which improves with increasing cork content. This phenomenon can be attributed to the closed-cell structure of cork, which traps non-flowing gases and reduces convective heat transfer [37]. Furthermore, the extensive surface area of the cork cell cavities elongates the heat transfer pathway along the cell walls, thereby impeding the heat transfer rate [38]. Moreover, the incorporation of cork creates interfacial gaps within the originally densely layered microstructure of the SNC material (Figure 4b–d), enhancing the overall porosity of the composite. Consequently, the combined effects of cork and interfacial gaps contribute to the outstanding thermal insulation properties of the SNC-C material. It is evident that an increased cork content enhances the barrier effect provided by its inherent pore structure and its induced layered cellulose gap structure, resulting in outstanding thermal insulation performance. In summary, the introduction of cork significantly enhances the thermal insulation performance of the SNC-C material.

### 3.3. Cork Introduced into SNC Boards to Enhance Water Barrier Properties

**Figure 6:**
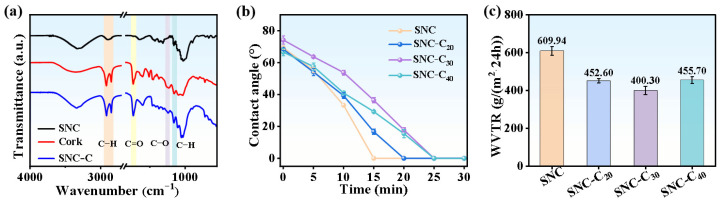
Water-repellent property of cork. (**a**) FTIR spectra of SNC fibers, cork particles, and SNC-C board; (**b**) variations of surface water contact angle with time and (**c**) water vapor transmission rate.

Suberin, the main component of cork, is a biopolyester rich in long-chain multifunctional fatty acids that provides strong water and solute barrier properties [39]. In comparison to pure SNC fibers, FTIR spectra showed that cork particles exhibit characteristic peaks at 2920 cm^−1^, 2850 cm^−1^, 1735 cm^−1^, 1235 cm^−1^, and 1150 cm^−1^, corresponding to the symmetric stretching vibration of C-H, asymmetric stretching vibration of C-H, C=O stretching vibration, C-O bending vibration, and C-O-C stretching vibration of the suberin component [40], respectively (Figure 6a). These peaks indicate that the cork used in this study contains suberin, which possesses the potential to enhance the water-repellent properties of the material [41]. In addition, the presence of all the characteristic peaks of the cork in the SNC-C board indicates the successful incorporation of the cork into the SNC board.

When water droplets were placed on the surface of SNC-C materials with varying cork contents, the initial static water contact angles were comparable, averaging around 70°. Over time, the water contact angle gradually decreased until the water droplets completely penetrated into the material, at which point the contact angle reached 0° (Figure 6b). It is important to note that the time required for the water droplets to penetrate the material varied depending on the cork content. For cork contents of 0%, 20%, 30%, and 40%, the corresponding penetration times were 15 min, 20 min, 25 min, and 25 min, respectively (Figure 6b). This indicates that the water droplet penetration time gradually extended, reaching a maximum at 30% cork content. However, further increasing the cork content did not prolong the water penetration time. These results clearly demonstrate that cork effectively delays the penetration of water droplets, thus enhancing water resistance. At a cork content of 30%, the surface structure of the material was relatively dense, with no apparent holes (Figure S4), and the internal cellulose structure was also quite dense (Figure 4c), with the visible cross-sectional pores primarily representing the cork cellular cavity (Figure S5). This dense fiber structure, combined with the barrier effect of cork, synergistically contributes to the optimal delay in water droplet penetration.

When water vapor passes through the material, the barrier effects vary with different cork contents. At 0% cork content, the WVTR of SNC was 609.94 g/(m^2^·24 h). As the cork content increased to 20%, 30%, and 40%, the WVTR decreased to 452.60 g/(m^2^·24 h), 400.30 g/(m^2^·24 h), and 455.70 g/(m^2^·24 h), respectively (Figure 6c). All these values were lower than that of the pure SNC material, indicating that cork provides varying degrees of water vapor barrier effects [37]. Overall, as the cork content increases, the material initially exhibited a decrease in WVTR followed by an increase. The lowest WVTR was observed at 30% cork content, showing a 34.4% reduction compared to the pure SNC material, demonstrating the most effective water vapor barrier performance. This phenomenon is also attributed to the optimal synergy between the densely layered fiber structure and the barrier properties of cork at 30% cork content (Figure 4c, Figures S4 and S5). However, further increasing the cork content to 40% induces more cracks in the layered fiber structure of the material, which adversely affects the water vapor barrier performance (Figure 4c).

In short, the results clearly demonstrate that cork significantly enhances the barrier properties of SNC-C material against both water droplets and water vapor.

### 3.4. Optimization and Evaluation of Cork Content

**Figure 7:**
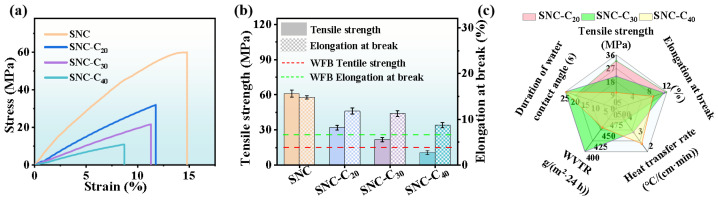
Optimization and evaluation of cork content. (**a**) Stress–strain curves and (**b**) tensile strength and elongation at break of SNC-C with varying cork content. (**c**) Comparison of the comprehensive performance of SNC-C materials as demonstrated by radar diagrams.

It is important to note that the excessive addition of cork induces interfacial gaps within the layered fiber structure of the SNC materials (Figure 4b–d). These interfacial gaps not only affect the overall water-repellent properties but also diminish the cohesion force of the molecular chains in the cellulose matrix, thereby negatively impacting the mechanical strength of the materials [42]. As the cork content increased from 0% to 20%, 30%, and 40%, the tensile strength of the material decreased from 60.93 MPa to 32.02 MPa, 21.67 MPa, and 10.73 MPa, respectively (Figure 7a,b). Similarly, the elongation at break decreased as the cork content increased (Figure 7a,b), indicating a corresponding reduction in the fracture toughness of the material due to the negative impact of matrix porosity on cohesion [42]. Notably, when the cork content reaches 30%, the tensile strength and elongation at break of the material (SNC-C_30_) remain superior to those of commercially available wood cellulose-fiber paper lunch boxes (Figure 7b), indicating the material meeting the market requirements for both strength and toughness in paper-based packaging materials.

A radar chart comparing the comprehensive performance (including tensile strength, elongation at break, heat transfer rate, WVTR, and water droplet penetration time) of materials with different cork contents shows that the material’s overall performance is optimal at a cork content of 30% (Figure 7c). Furthermore, the mechanical strength of SNC-C_30_ was compared with other previously reported biomass packaging materials, as shown in Table 1.

## 4. Conclusions

In summary, this study developed a novel sustainable paper-based packaging material (SNC-C_30_) with high strength, thermal insulation, and water resistance properties using agricultural waste straw and forestry waste bark. The high aspect ratio (~250) of SNC provided the material with high mechanical strength, demonstrated by a tensile strength of SNC boards of 60.93 MPa. Additionally, the unique honeycomb-like closed-cell structure of the cork particles reduces the material’s heat transfer rate (2.90–3.01 °C/(cm·min)) by 35.95–40.82%. The combined factors of the closed-cell structure and suberin component of the cork collectively provide the material with excellent water-resistance properties, evidenced by a WVTR of SNC-C_30_ of 400.30 g/(m^2^·24 h). Therefore, the SNC-C_30_ material developed in this study possesses excellent mechanical strength, thermal insulation, and water resistance, providing a new perspective for the development of fully green and sustainable paper-based multifunctional packaging materials.

## Figures and Tables

**Table 1:** Mechanical strength of other biomass packaging materials.

Materials	Tensile Strength (MPa)	Elongation at Break (%)	References
BC/CMC	19.64	4.61	[43]
GEL/NCS/GLY	8.67	11.01	[44]
PS/SP	7.02	6.82	[45]
CH	1.09	—	[46]
LVB-F	15.3	0.8	[47]
SNC-C_30_	21.67	12.28	Our work

BC/CMC, bacterial cellulose/carboxymethyl cellulose film; GEL/NCS/GLY, gelatin/native corn starch/glycerol film; PS/SP, plasticized starch/soy pulp film; CH, chitosan film; LVB-F, vascular bundles film.

## Data Availability

Data are contained within the article and supplementary materials. Further inquiries can be directed to the corresponding author.

## Supplementary Materials

The following supporting information can be downloaded at https://www.mdpi.com/article/10.3390/polym17010006/s1: Figure S1: 3D AFM morphology of SNC fibers; Figure S2: SEM morphology of the inner surface of SNC board; Figure S3: Optical microscopic morphology of the fracture surface of the SNC board (demonstrating ductile tearing morphology as shown in the red dashed area); Figure S4: Surface SEM morphology of SNC-C_30_; Figure S5: Cross-section SEM morphology of SNC-C_30_.
