# Supplementary material for: A Full Green, Sustainable Paper-Based Packaging Material with High-Strength, Water Resistance, and Thermal Insulation
Source: Polymers (Basel). 2024 Dec 24;17(1):6. doi: 10.3390/polym17010006 (PMC11723104; doi:10.3390/polym17010006)
Supplement: Supplementary file 1 [file polymers-17-00006-s001.zip › polymers-3363709-supplementary.pdf]

Supplementary Material

A Full Green, Sustainable Paper-based Packaging Material with High-Strength, Water Resistance, and Thermal Insulation

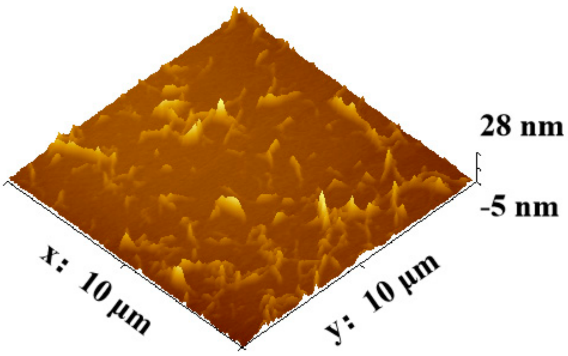

Figure S1. 3D AFM morphology of SNC fibers.

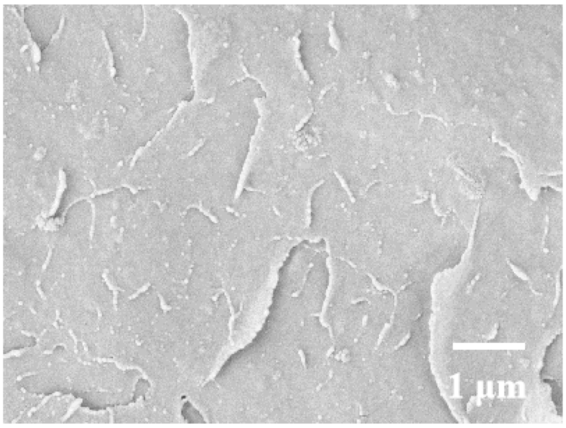

Figure S2. SEM morphology of the inner surface of SNC board.

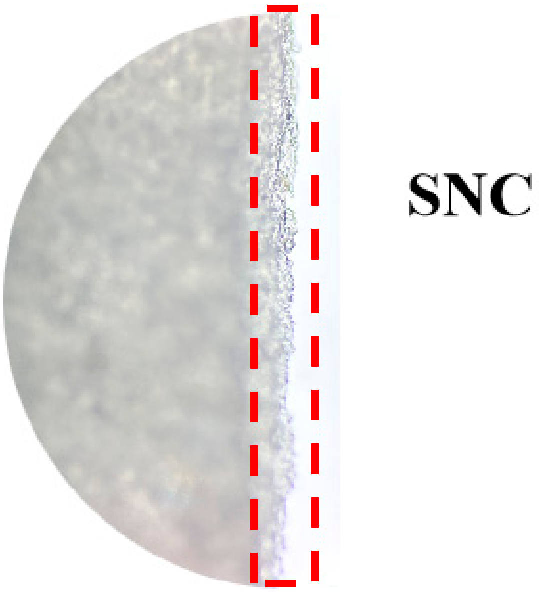

Figure S3. Optical microscopic morphology of the fracture surface of SNC board (demonstrating ductile tearing morphology as shown in the red dashed area).

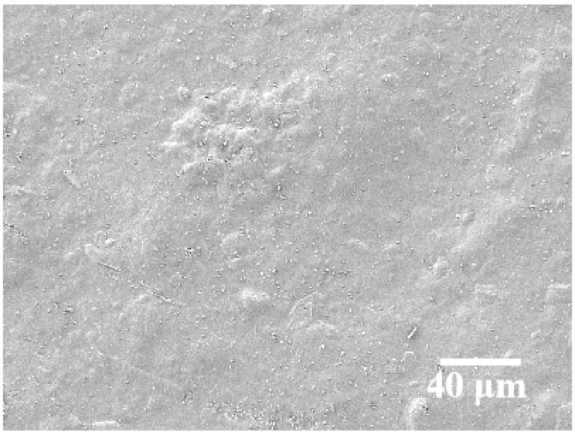

Figure S4. Surface SEM morphology of SNC−C_{30}.

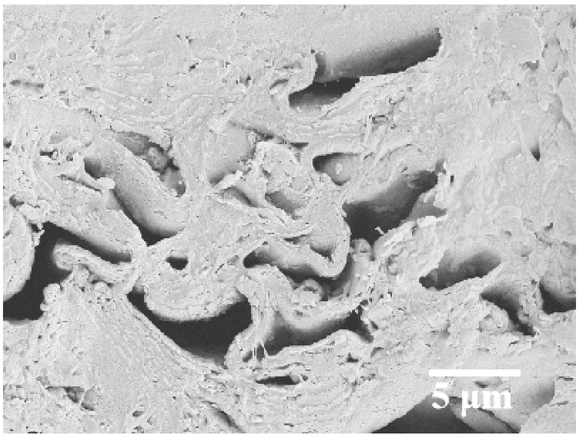

Figure S5. Cross-section SEM morphology of SNC−C_{30}.
